# Experimental and theoretical aspects of the growth of vertically aligned CNTs by CCVD on AZO substrate

**DOI:** 10.1038/s41598-024-57862-w

**Published:** 2024-03-27

**Authors:** Lilla Nánai, Zoltán Németh, George Kaptay, Klara Hernadi

**Affiliations:** 1 Institute of Physical Metallurgy, Metal Forming and Nanotechnology, University of Miskolc, Miskolc, 3515 Hungary; 2 Advanced Materials and Intelligent Technologies Higher Education and Industrial Cooperation Centre, University of Miskolc, Miskolc, 3515 Hungary; 3 REN-HUN-ME Materials Science Research Group, Egyetemvaros, Miskolc, 3515 Hungary; 4 Department of Applied and Environmental Chemistry, University of Szeged, Szeged, 6720 Hungary

**Keywords:** Vertically aligned carbon nanotubes, Aluminum doped zinc oxide, Catalytic chemical vapor deposition, CNT growth mechanism, Nanoscale materials, Nanoscience and technology, Materials chemistry

## Abstract

An efficient and reproducible growth of vertically aligned carbon nanotubes by CCVD requires accurate and specific setting of the synthesis parameters and the properties of catalyst thin layers. In this work, the growth of vertically aligned carbon nanotubes onto AZO (= aluminum doped zinc oxide) glass substrate covered by Al_2_O_3_ and Fe-Co catalyst layer system is presented. Investigation of the effect of catalyst composition and synthesis temperature on CVD growth revealed the optimum condition of the synthesis. The analysis of as-prepared samples by SEM, TEM and Raman spectroscopy was carried out to prove the structure and quality of carbon deposit. Theoretical considerations have supported speculative ideas about the role of the support layer, the transformation of the catalyst layer in the presence of hydrogen gas and the growth mechanism of carbon nanotubes. The mechanism of CNT growth is modelled and the order of magnitude of experimentally observed vertical linear growth rate of CNT (several nm/s) is reproduced.

## Introduction

Carbon nanotubes (CNTs) have gained a lot of attention over the last 3 decades due to their prominent chemical, physical and electrical properties, they play important roles in electrical, mechanical developments and additives in composites as well^1^. Since the first synthesis of vertically aligned carbon nanotubes (VACNTs) in 1996^2^ have become an important, worldwide subject of study due to this material formulation has been used as a cutting-edge architectural design to incorporate into numerous nanotechnology-related devices. Based on the current state of the science, catalytic chemical vapor deposition (CCVD) based process is the most suitable method^3^ to prepare VACNTs on various substrates using a variety of different synthesis parameters according to the intended use^4^. The CVD parameters have been established well enough in the past years to control the growth of CNTs, but the properties of catalyst and support oxide layer system can sensitively influence the growth of VACNTs, for example, orientation, length, density, crystalline or graphitic properties etc^5–9^.

The growth mechanism of carbon nanotubes is still a rather contradictory topic. There are some acknowledged theories about the growth of carbon nanotubes by researchers^10–16^, in particularly, a) the process begins with the formation of catalyst nanoparticles from the originally homogeneous catalyst layer (in the presence of hydrogen gas and in the absence of carbon source), followed by their reduction and continues with the nucleation and the growth of carbon nanotubes. Furthermore, there are two generally accepted growing mechanism models for the growth of carbon nanotubes^17,18^, the root growth and the tip growth, which are related to the interaction between the catalyst particles and the substrate or the catalyst support. In the case of root growth mechanism, due to nanoparticles’ strong attachment to the support, catalyst particles stay at the surface and contributing to the growth of carbon nanotubes, while in the case of tip growth mechanism, due to weak interaction between the nanoparticles and substrate, the catalyst particle is located at the “tip” of the carbon nanotube, its activity decreases and ceases completely when it is completely covered by carbon layer. The oxide layers (Al_2_O_3_, TiO_2_, SiO_2_ etc*.*) on substrates play a significant role in the synthesis of CNTs, by contributing to the transformation of homogeneous metallic layer (Fe, Co, Ni and their alloys) to individual active nanoparticles and their uniform distribution on the substrate surface^19–22^.

Transparent conductive oxides (TCOs) have drawn increasing attention in various research^23–25^. The most prominent TCOs are indium tin oxide (ITO), fluorine tin oxide (FTO) and aluminum doped zinc oxide (AZO). ITO was popularly used for various TCO applications due its suitable features^26^. In recent years due to the shortage of the rare-earth indium, the natural brittleness and high manufacturing costs of ITO have limited the mass production of flexible and low-cost devices^27–30^.

Particularly, transparent conductive oxide thin films have various applications in opto-, microelectronic and photovoltaic devices, such as sensors, solar cells, LEDs, display panels etc*.* due to their superb electrical conductivity and optical transparency^31–33^.

However, ITO and FTO glass substrates are the most used and well known among TCO substrates, but their applications are rather limited for VACNT growth due to their temperature sensitive properties^23^. ITO glass electrical conductivity drastically decreases during calcination or heat treatment above 350–450 °C, while FTO shows similar changes above 550 °C^34,35^. In the literature no results were found about aluminum doped zinc oxide (AZO) coated glass substrates suitability for VACNT growth.

Unfortunately, few works can be found, which are focusing on the thermodynamic calculations of aligned carbon nanotubes. There are various factors that can control the efficiency of growth of carbon nanotubes^36^. The growth rate during the synthesis can be limited by synthesis temperature, catalyst quality and concentration, the type of gas feedstock, surface reactions on the catalyst particle, the carbon diffusion through the catalyst particle bulk phase or over the surface of the catalyst particle. The growth mechanism of carbon nanotube is very complex, involving processes such as dissociation of carbon source and diffusion of carbon through or over the catalyst nanoparticles^37^. The dissociation of carbon source and the rate of the dissociation can influence the formation of carbon nanotubes, which mainly depends on properties such as Gibbs free energy and formation enthalpy^38^. However, none of the theoretical approaches published until now describes the system applied in this study.

An efficient and reproducible growth of vertically aligned carbon nanotubes by CCVD requires accurate and specific setting of the synthesis parameters and the properties of catalyst thin layers. In this work, we demonstrate the growth of vertically aligned carbon nanotubes, to the best of our knowledge, onto AZO glass substrate with and without Al_2_O_3_ layer and Fe-Co catalyst layer system, which have not been used in previous research. In addition to the experimental approach, our aim was to gain a deeper understanding of the behavior of the substrate (the alumina) and the catalyst layer under the reaction conditions used, and we have therefore supported our results with thorough theoretical considerations as well.

## Experiments

### Materials

Aluminum doped Zinc Oxide coated soda lime glass (TECHINSTRO) was used as substrate for the synthesis. Cobalt (cobalt(II) nitrate hexahydrate, 99%, Sigma-Aldrich), iron (iron(III) nitrate nonahydrate, 99.9%, Sigma-Aldrich), and aluminum (aluminum(III) nitrate nonahydrate, 99.9%, Sigma-Aldrich) precursors dispersed in absolute ethanol (VWR) were used to prepare the subsequent support and catalyst layers. During the syntheses, nitrogen (99.995%, Messer) was used as the carrier gas, ethylene (> 99.9%, Messer) as the carbon source, and hydrogen (99.5%, Messer) as the reducing agent.

### Catalyst preparation

**Figure 1:**
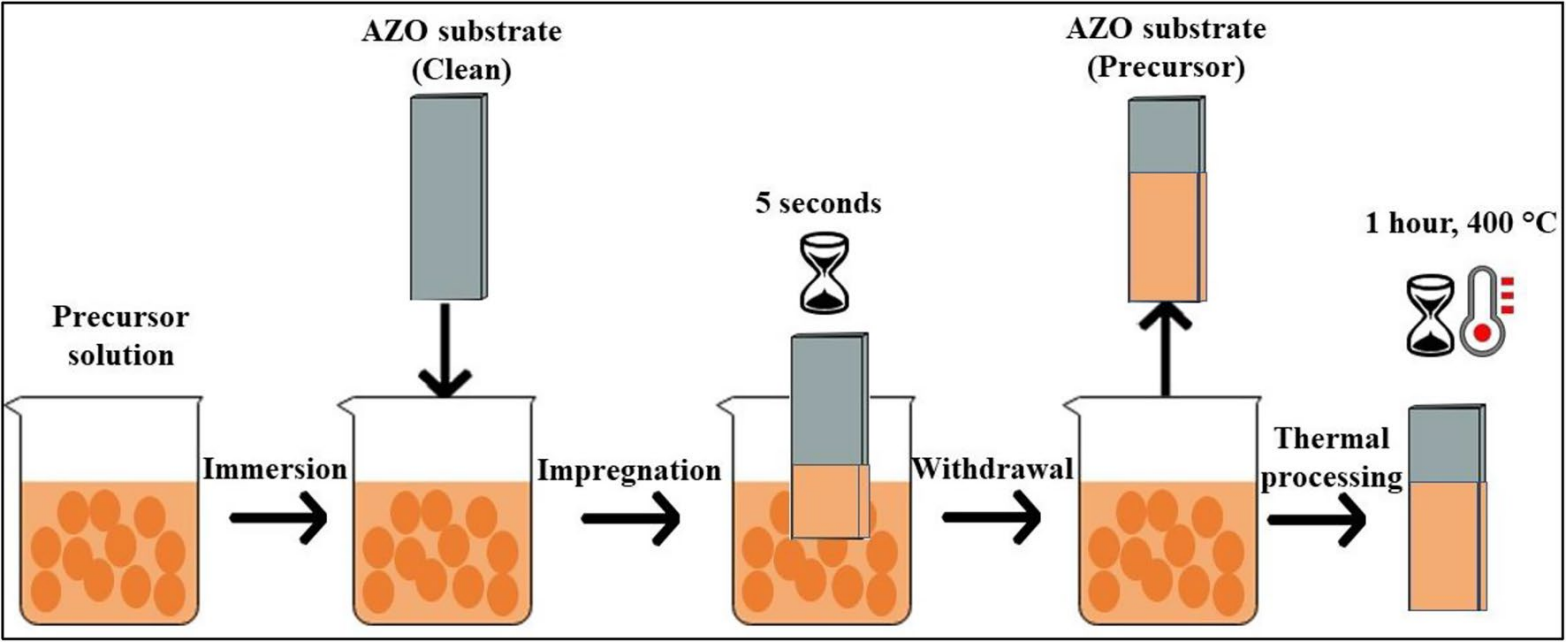
Schematic figure of the preparation of the catalyst and support layer via dip coating technique.

A simple and cheap method, dip coating was used to prepare catalyst layer on the surface of the AZO substrate. The schematic illustration of sequential steps can be seen in Fig. 1. Firstly, the substrates were cut to size (5 × 25 mm), then rinsed with isopropanol, distilled water, and acetone to remove potential contaminants from the surface. For dip coating the catalyst solution was prepared by mixing Fe and Co precursors at different molar ratios (0:1, 1:3, 2:3, 1:1, 3:2, 3:1 and 1:0) by dissolving Co(NO_3_)_2_ × 6H_2_O and Fe(NO_3_)_3_ × 9H_2_O precursors in absolute ethanol at their total concentration of 0.11 M respectively. The catalyst solutions were freshly prepared to avoid undesirable component formation and decomposition. The substrates with catalyst layer were heat treated in a static oven at 400 °C for 1 h to stabilize the catalyst precursor particles on the surface.

The dip coating method was also used for the Al_2_O_3_ support oxide layer preparation, respectively. Al(NO_3_)_3_ × 9H_2_O precursor was dissolved in absolute ethanol at a concentration of 0.11 M. After this step it was heat treated in static oven at 400 °C for 1 h to stabilize and form Al_2_O_3_ support oxide layer on the substrate, which was followed by catalyst deposition on the surface.

### CCVD synthesis

To perform CCVD synthesis, substrates with catalyst layers were placed into a horizontal quartz tube reactor (diameter 20 mm, length 80 mm) at 600, 650 and 700 °C. The reaction time was 30 min, which was preceded by a 5-min nitrogen (50 cm^3^/min) rinse and the samples were treated by an additional 5-min reduction with hydrogen (50 cm^3^/min), then ethylene (70 cm^3^/min) was introduced to the system to start the growth of vertically aligned carbon nanotubes. At the same time, as the ethylene was launched into the system, nitrogen gas (25 cm^3^/min) on a side branch was bubbled through 25 °C distilled water to increase the efficiency of the synthesis by adding water vapor to the reactor. Even after the carbon source and water vapor were introduced, hydrogen and nitrogen gases were continuously fed into the CVD system during the entire synthesis.

No deformation or change of material properties of glass substrate was observed during or after the whole process.

Orientation of the CNTs was observed by a Hitachi S-4700 field emission cathode scanning electron microscope (FESEM) in secondary electron imaging mode. During the SEM measurements, the samples were tilted at an angle of 35° to reveal the sides as well as the top of the carbon nanotubes, from which we could later determine the height of the VACNTs using Image J software to draw further conclusions about their structure. The synthetized samples were examined by FEI Tecnai G2 20X-Twin-type transmission electron microscope (200 keV). The morphology and structure of the nanomaterials has been analyzed and diameter of carbon nanotubes have been measured. The samples were prepared by dropping the absolute ethanolic suspension of carbonaceous material on 300 mesh copper grids (Ted Pella Inc.). Raman spectra of the samples were recorded using a Thermo DXR Raman microscope in the 50–1860 cm^−1^ range with ~ 3 cm^−1^ resolution. The excitation wavelength was 532 nm. The spectra were taken by 5 mW laser power, which was focused onto the nanotubes by a 50 × magnification microscope objective for 2·10 s.

## Experimental results and discussion

Based on incomplete information in the scientific literature about VACNT synthesis on AZO glass, the experiments were conducted on three different synthesis temperatures, seven different Fe:Co catalyst ratios, also in the presence of Al_2_O_3_ layer and without Al_2_O_3_ layer to investigate the effect of synthesis parameters on the properties of VACNTs. The sample preparation and the CCVD parameters were the same for each experiment as it was mentioned in the Experiments section. The synthesis temperatures of 600, 650 and 700 °C were chosen because, based on our preliminary experiments, no carbon deposition was observed on the AZO substrate surface below 600 °C via CCVD method.

Monometallic catalyst layers, both iron and cobalt have been reported to have significant activity as catalysts in carbon nanotubes production via the CVD method^20,39,40^.

**Figure 2:**
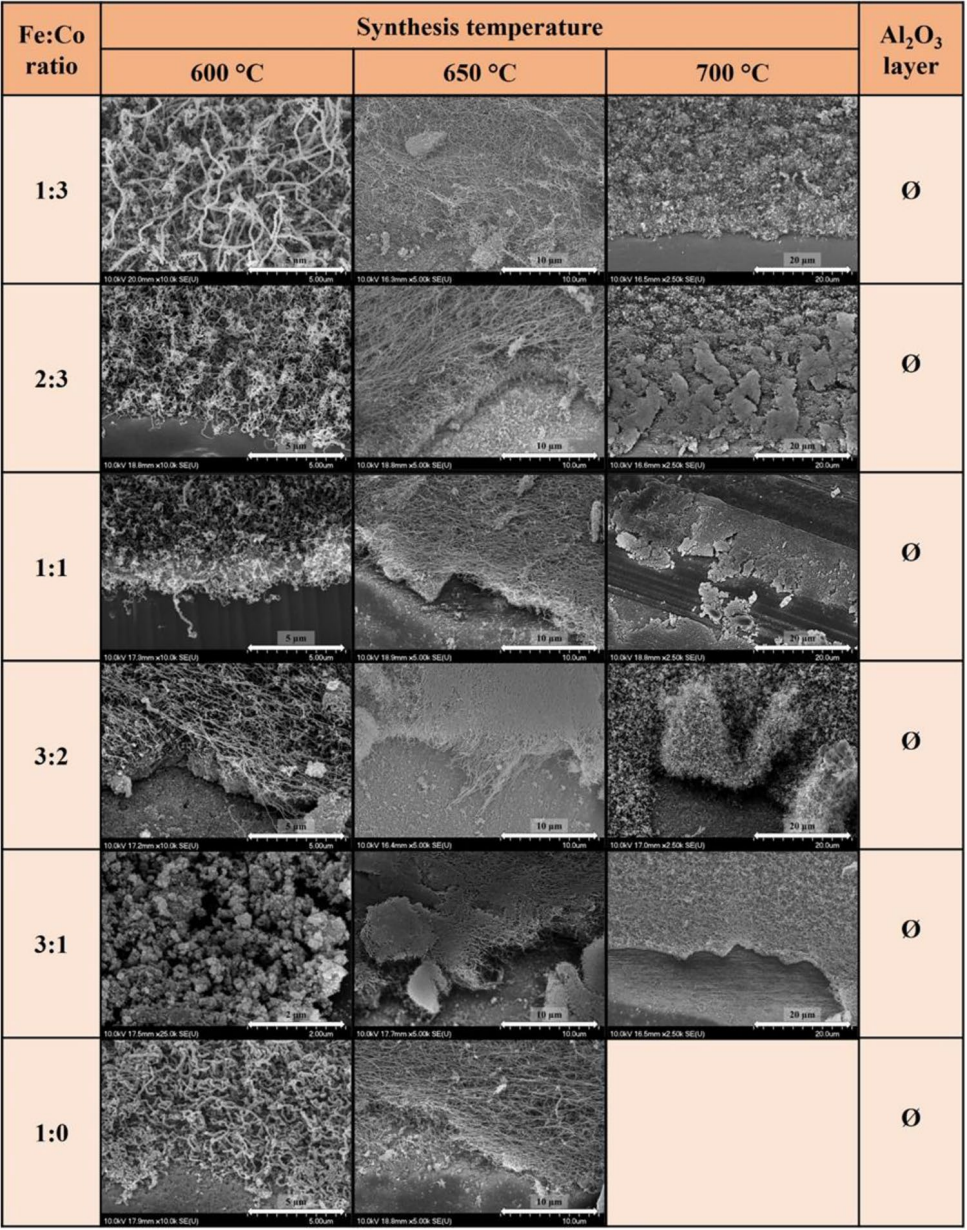
SEM images of VACNTs synthesized on AZO substrate without Al_2_O_3_ layer at 600 °C, 650 °C and 700 °C temperatures with different Fe:Co ratios.

However, in our synthesis system no carbon deposition was observed in case of using only pure cobalt as a catalyst. Similar results were obtained for the pure iron catalyst at 700 °C both in the presence and absence of the support layer, and neither carbon deposition nor CNT growth was significant for the pure iron catalyst at other temperatures. The conclusion is that bimetallic catalyst layer is essential to synthetize VACNTs on the surface of AZO substrate. In the first major experimental block the formation of carbon deposit was investigated as a function of reaction temperature and catalyst composition in the absence of Al_2_O_3_ layer (Fig. 2). The following conclusions can be drawn from these results: at 600 °C mainly amorphous carbon, carbon fibers and coiled CNTs were observed, at higher temperatures, CNTs were formed irregularly in larger bundles and were observed in agglomerates on the surface of the AZO substrate. At 650 °C and 700 °C synthesis temperatures, mainly carbon nanotubes were observed on the AZO surface. However, similar conclusions could be drawn as at 600 °C, that the carbon nanotubes were not arranged vertically and were observed in bundles. From the analysis of the SEM images, the following can be concluded: the highest amount of carbon deposition was achieved at 650 °C, covering almost the entire surface of the substrate, however, in most cases the carbon deposition was still achieved only in spots, which might be caused by the surface properties of the AZO substrate which affected the formation of the catalyst layer by dip coating, limiting the formation of a uniform layer (see also Fig. 5).

Interesting fact, that large quantities of coiled CNTs were observed at Fe:Co ratios of 1:3, 2:3, 1:1 and 1:0 at 600 °C synthesis temperature (Fig. 2). In the literature, the formation of carbon nanotubes with a helical structure is explained by the periodic incorporation of pentagon and heptagon pairs into the predominantly hexagonal carbon structure, resulting in curved surfaces^41–43^.

**Figure 3:**
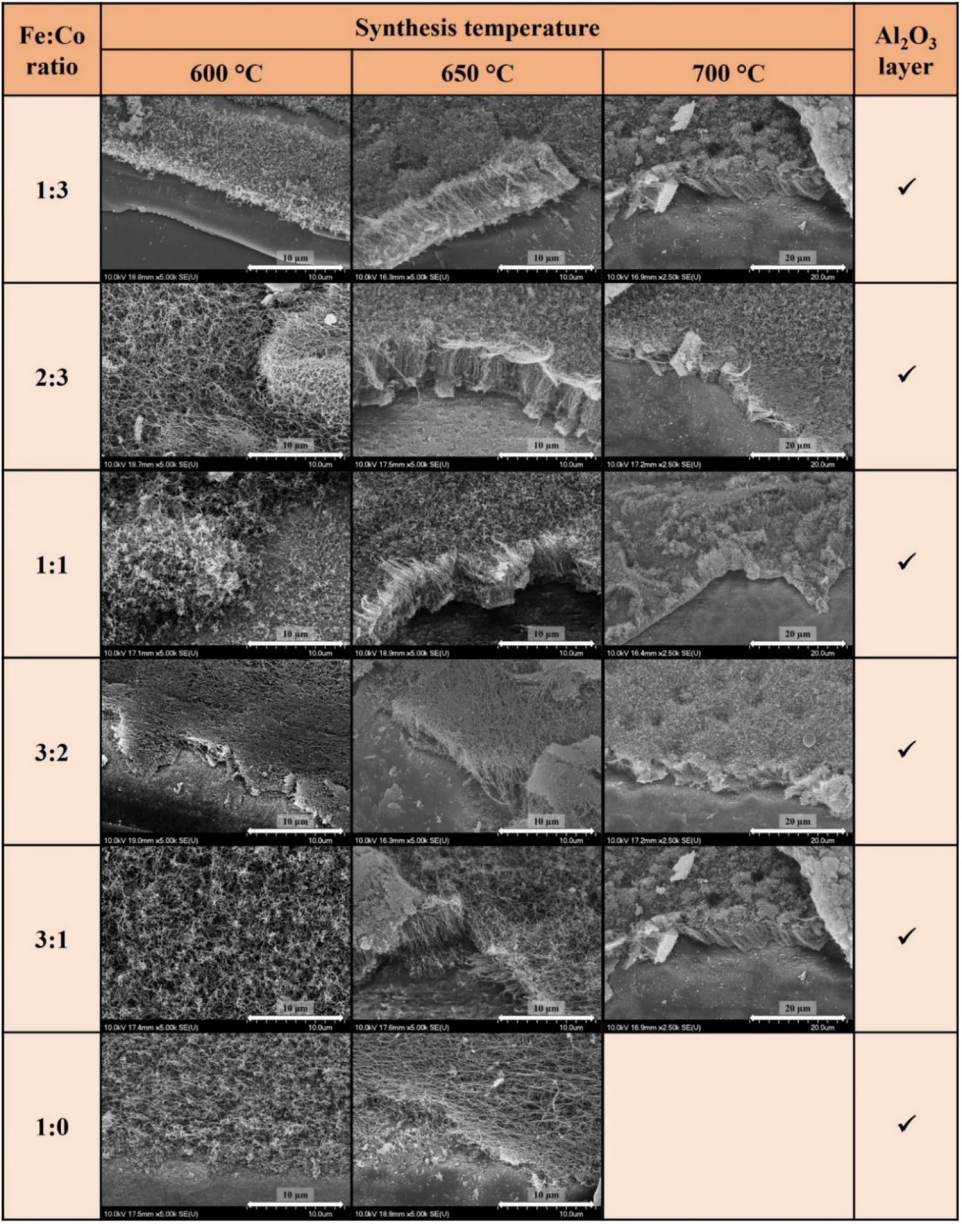
SEM images of VACNTs synthesized on AZO substrate with Al_2_O_3_ layer at 600 °C, 650 °C and 700 °C temperatures at different Fe:Co ratios.

In the second larger block of experiments, the experiments described above were repeated, with the modification that the Al_2_O_3_ support layer was first deposited by dip coating before the catalyst layer was prepared. Analysis of the SEM images (Fig. 3) showed a significant improvement at all three synthesis temperatures in the presence of the Al_2_O_3_ support layer, with clear formation of carbon nanotubes, although at 600 °C the carbon nanotubes were still not aligned. At 650 °C, carbon nanotubes were clearly observed vertically aligned on the AZO substrate surface at all catalyst ratios and at 700 °C, apart from the pure iron catalyst, also appeared vertically aligned carbon nanotubes.

**Figure 4:**
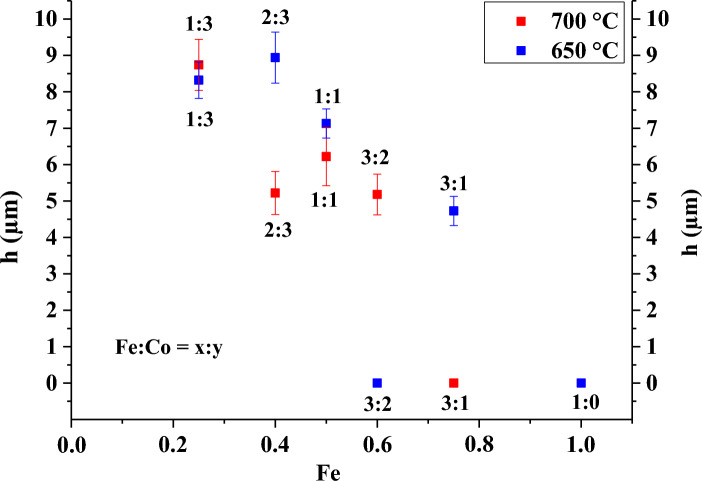
Height of VACNTs synthetized on AZO substrate with Al_2_O_3_ layer by varying Fe:Co catalyst ratio at 650 °C and 700 °C temperatures.

The SEM image was used to determine the height of the carbon nanotubes synthesized at 650 °C and 700 °C (Fig. 4), and it was observed that the height of the carbon nanotubes decreased with increasing temperature for the same catalyst composition. The height at the catalyst composition of 3:2 at 650 °C could not be determined from the SEM image, but it can be assumed that following the trend stated before, the carbon nanotubes at this ratio are higher than at 700 °C. In contrast to other studies^44–48^, where the aim was to produce higher VACNTs, in the present work short carbon nanotubes were grown, with the advantage of short carbon nanotubes having better thermal conductivity^49^. It was revealed that the highest VACNTs (9.0 µm and 8.7 µm) were obtained in case of Fe:Co 2:3 at 650 °C and Fe:Co 1:3 at 700 °C, which is an additional difference from the published results is that the highest carbon nanotubes were not formed at the 1:1 catalyst composition^50^.

**Figure 5:**
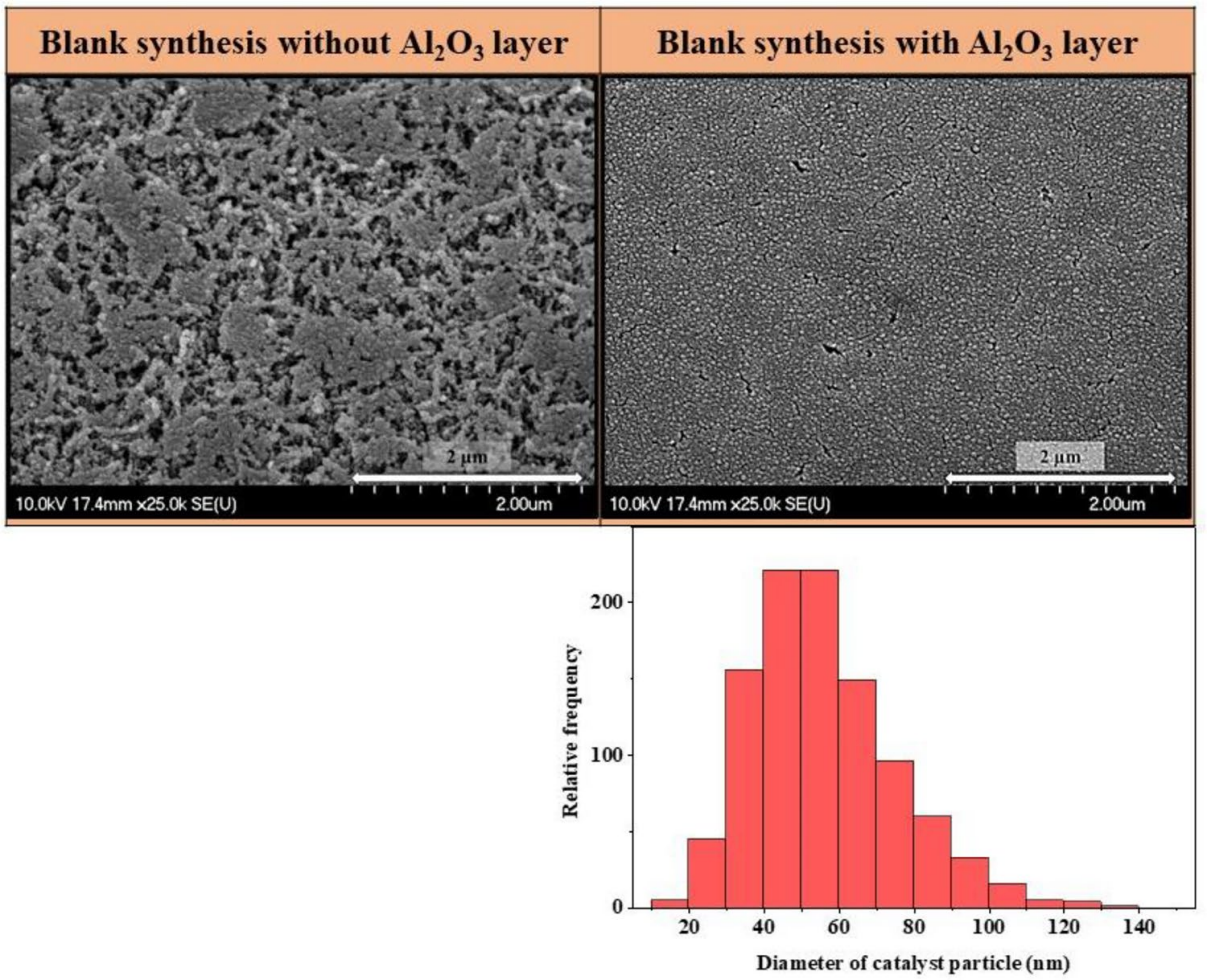
SEM images of blank synthesis without and with Al_2_O_3_ layer, and diameter distribution of catalyst nanoparticles based on the images.

Applying Al_2_O_3_ layer was beneficial during the synthesis as it presumably inhibits the aggregation and diffusion of iron and cobalt catalyst particles, which ensured that the catalyst nanoparticles were evenly distributed on the surface. This claim was verified by carrying out blank experiments as well, in which hydrogen gas was circulated in the system for 5 min, without carbon source. The experiments were carried out both with and without Al_2_O_3_ support layer (Fig. 5). Since, regardless of the temperature and catalyst composition used, a clear change in the presence of the support layer was observed during the blank synthesis, only SEM images of samples prepared at 650 °C with a Fe:Co 3:1 catalyst ratio are showed as representative.

Based on the SEM images it was observed that without support layer aggregates, catalyst “islands” were formed on the surface of AZO, while in the presence of Al_2_O_3_ layer, smaller individual catalyst particles were formed in close proximity to each other, hence allowing the formation of VACNTs. In general, the catalytic activity and lifetime of catalyst particles are affected by the diffusion of catalyst nanoparticles on the surface of substrate and in the support layer^51^.

**Figure 6:**
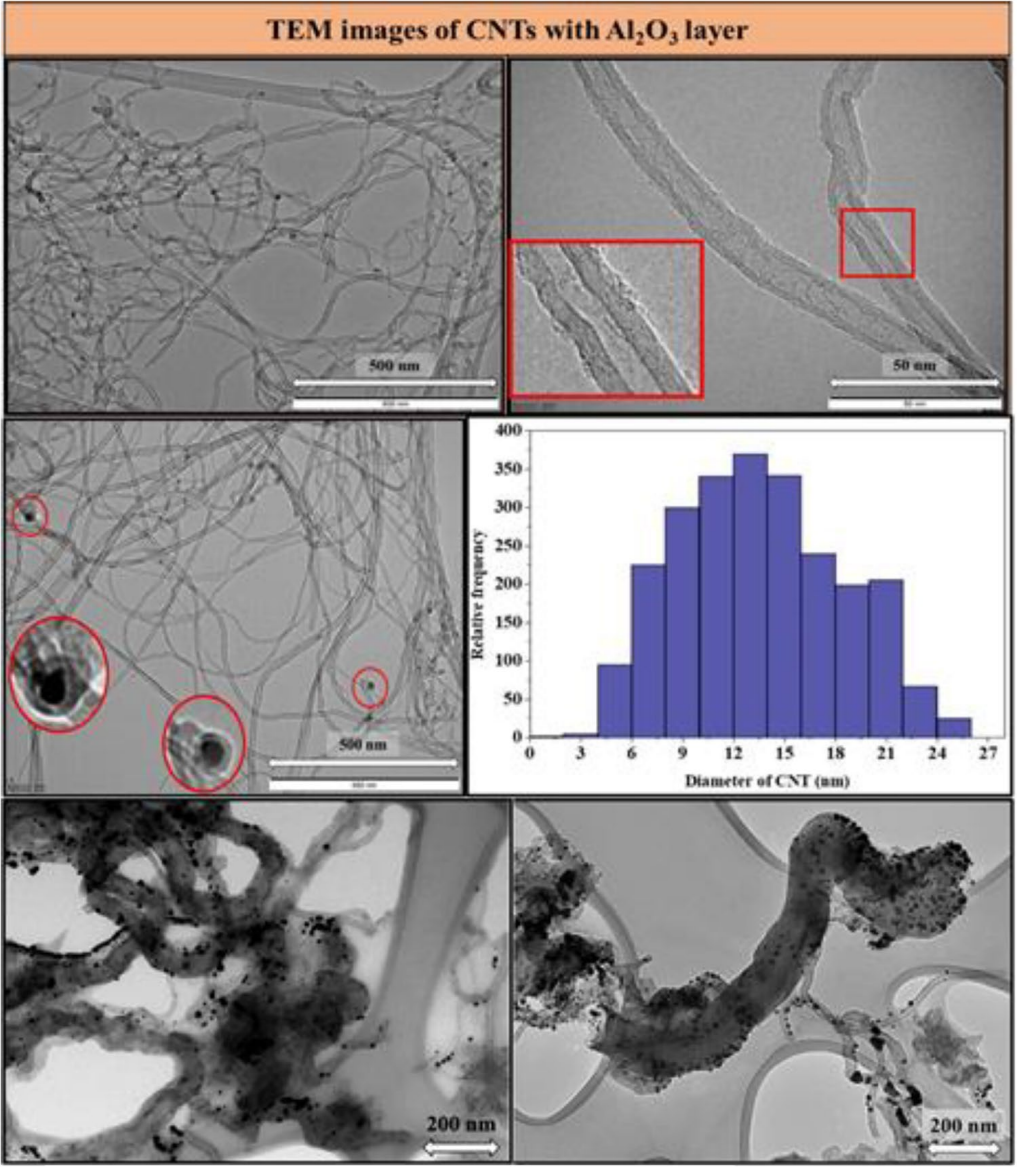
TEM images of CNTs with different magnifications, diameter distribution of CNT based on the images and TEM images of carbon fibers.

To confirm graphitic nature, samples synthesized in the second experimental block were characterized by TEM as well (Fig. 6). Since there is no significant difference in the quality of carbon nanotubes synthesized with different catalyst compositions, the TEM images are representative (650 °C, Fe:Co ratio 2:3).

**Figure 7:**
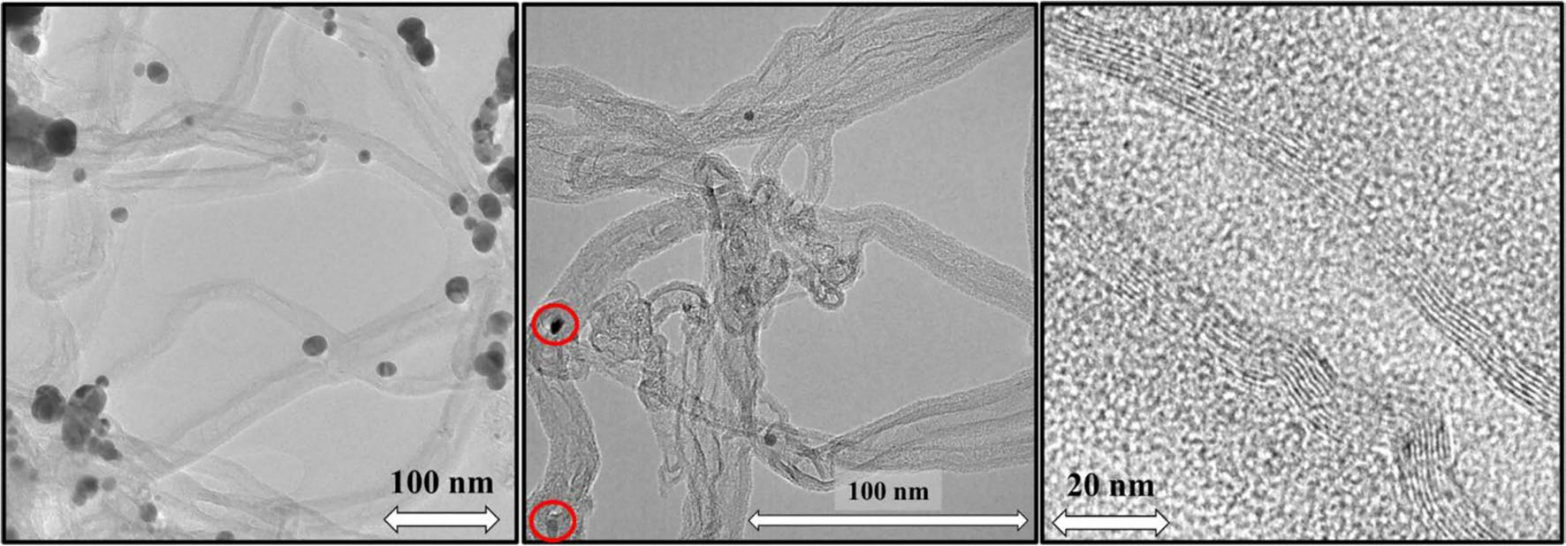
TEM and HRTEM image of carbon nanotubes with amorphous carbon coverage of carbon nanotubes. (Note: red circles highlight catalyst nanoparticles in the carbon nanotubes, while the other nanoparticles are gold nanoparticles from the preparation of samples for SEM measurement).

Based on the TEM images, the following conclusions could be drawn: multi-walled carbon nanotubes were formed during the CCVD process, where the number of walls varied from ~ 7 to 10, with some irregularity in the walls (shown with a red square in the images), which suggests that the carbon nanotubes contain many defects sites in the structure, which indirectly predicts that the graphitic and conductive properties of carbon nanotubes might be average. Moreover, only few abundances of catalyst nanoparticles were visible in certain regions of TEM images (shown with red circles), which suggests that root growth mechanism was dominant during the CCVD, and the surface of carbon nanotubes contains only a small amount of amorphous carbon layers. This latter fact is probably due to introduction of water vapor into the system which prevents not only the early inactivation of the catalyst particles but also the formation of undesired carbon forms in general. The outer diameter of carbon nanotubes ranged from 6 to 20 nm. The last two TEM images, which represents samples prepared during the second experimental series with Al_2_O_3_ support layer on the surface of AZO at 600 °C. CNTs were visible on the SEM images of Fig. 3, however the vertically alignment have not occurred. On the TEM images of these samples, it was observed, that thick carbon fibers were grown in the surrounding of carbon nanotubes. The small particles seen in the images are not cobalt and iron catalyst nanoparticles as measured by TEM EDS, but gold nanoparticles which were deposited before SEM measurement on the sample. The TEM and HRTEM images in Figs. 6 and 7 confirmed the growth of multi-walled carbon nanotubes. In the case of the samples where carbon deposition was detected and irregularly structured carbon nanotubes were formed (Fig. 3), the HRTEM images in Fig. 7 revealed high amounts of amorphous carbon deposited on the outer walls of the carbon nanotubes and carbon fibers in the surrounding environment. Based on these results, future experiments will focus on the effect of water vapor during CCVD syntheses and on optimizing the amount of water vapor in the system to reduce the quantity of amorphous carbon and improve the quality of carbon nanotubes.

**Figure 8:**
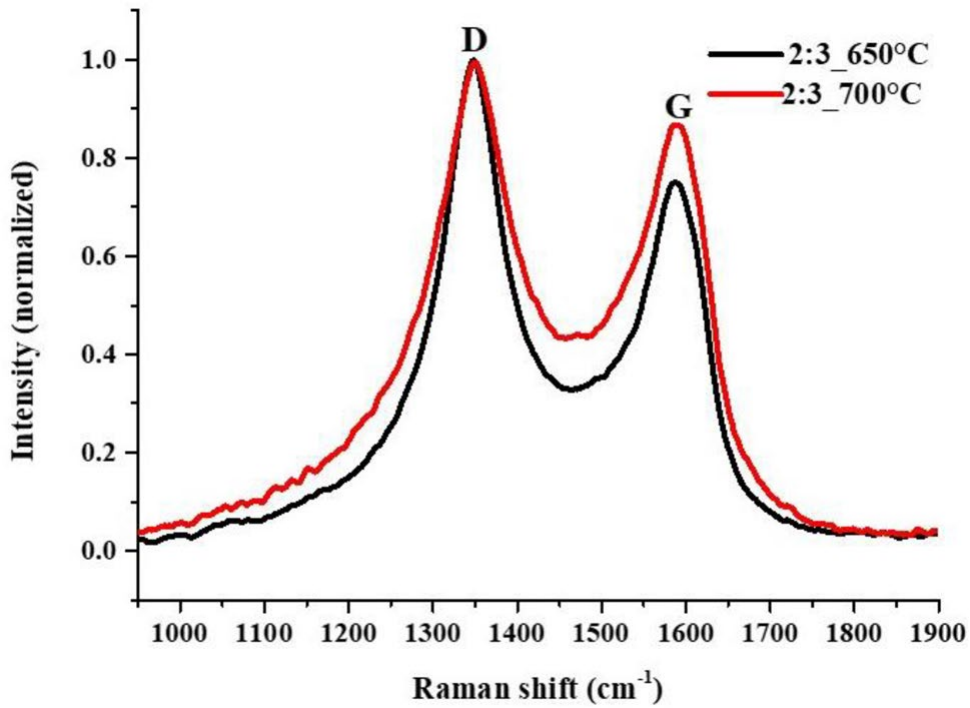
Raman spectra of VACNTs synthetized in the presence of Al_2_O_3_ support layer on AZO substrate at 650 °C and 700 °C.

Raman spectroscopy was also used to characterize the samples that were prepared in the second experimental block. Representative Raman spectra of VACNT samples prepared by 2:3 iron cobalt ratio at 650 °C and 700 °C synthesis temperatures, respectively (Fig. 8). According to previous research on Raman spectroscopy of CNTs, the ratio I_G_/I_D_, which must be less than 0.45 for high structure quality of the CNTs, determines how many defects there are in CNTs^52–54^. Their spectra are effectively very similar, which means that there is no significant difference in the quality of the carbon nanotubes, however, the sample synthesized at higher temperature has a slightly better I_G_/I_D_ ratio, which confirms the claim that the graphitic property of carbon nanotubes improves with increasing temperature^55^. Their I_G_/I_D_ ratios are ~ 0.87 ± 0.2 and ~ 0.75 ± 0.3, from which it can be concluded that carbon nanotubes have a less ordered structure with relatively high number of defects, their graphitic properties are not outstanding, which correlate well with the conclusions drawn from SEM and TEM images.

It can be concluded that the synthesis of VACNTs on AZO requires Al_2_O_3_ support layer and bimetallic catalyst layer where the beneficial parameters are 650 °C synthesis temperature with 1:3 and 2:3 catalyst compositions.

## Theoretical part

### Dissociation of nitrate precursors into oxides

Unfortunately, the standard thermodynamic functions of the relevant nitrate compounds are not known^56^, usually suggesting that these compounds are unstable. Indeed, Co(NO_3_)_2_ × 6H_2_O loses its crystalline water at 55–74 °C and decomposes to CoO at around 100 °C^57–59^. Also, Fe(NO_3_) × 9H_2_O spontaneously transforms to Fe_2_O_3_ near 125 °C^60,61^. Further, Al(NO_3_)_3_ × 9H_2_O decomposes spontaneously first to aluminum hydroxides (typically trihydroxides (Al(OH)3) and oxide-hydroxides (AlO(OH)) and then to Al_2_O_3_ starting from 150 °C^62–64^. Consequently, annealing the nitrate precursors at 400 °C for 1 h indeed ensures the formation of the desired oxides, which was our goal in this paper.

### Reduction of oxides in gaseous hydrogen

After preliminary heat treatment at 400 °C, layers on the surface of AZO substrate contain Co and Fe oxides, as well as alumina in selected samples. The Fe–Co catalyst is formed in hydrogen in the range 600–700 °C. Whether H_2_ gas decomposes an oxide or not depends on the temperature of the furnace and on the actual value of the p_H2O_/p_H2_ ratio, i.e. the ratio of the partial pressure of the water vapor formed to that of the residual hydrogen in our furnace.

Now, let us estimate the maximum p_H2O_/p_H2_ ratio that was present in our furnace supposing an oxide is reduced to a metal by hydrogen, and the released oxygen formed water vapor with hydrogen. First, let us estimate the volume of the oxide layer to be reduced. It is the surface area of the coated surface (0.5 cm × 0.5 cm) times the average thickness of the oxide layer (about 10 nm = 1 E-6 cm^65^) = 2.5 E-7 cm^3^. The order of magnitude of the molar volumes of oxides is 10 cm^3^/mol. Thus, the initial amount of oxide in the chamber is about 2.5 E-8 mol. Therefore, the approximated amount of water vapor that can form accompanying the full reduction of these oxides has the same magnitude of 2.5 E-8 mol. On the other hand, the volume rate of H_2_-feed into the furnace was 50 Ncm^3^/min for 30 min, i.e. total 1500 Ncm^3^ = 1.5 E-3 Nm^3^ of hydrogen passed the system. From the ideal gas law this volume corresponds to 0.062 mol of hydrogen. Therefore, the average p_H2O_/p_H2_ ratio in our experiments was 4 E-7 supposing for simplicity that the oxide was fully reduced, and this process had the same reduction rate during the 30 min treatment. If the equilibrium value of the p_H2O_/p_H2_ ratio for the reduction of a given oxide is much higher than the above value of 4 E-7, then the reduction of that oxide will take place and vice versa. The equilibrium p_H2O_/p_H2_ ratio for different reactions of interest at different temperatures of interest are calculated from standard thermochemical data of^56^ in Table 1. The following conclusions can be made from Table 1:

- the lowest equilibrium p_H2O_/p_H2_ ratio accompanying the reduction of CoO to Co is 33.5, being 8.4 E7 times larger than the average experimental p_H2O_/p_H2_ ratio of 4 E-7 found above, i.e. the reduction of CoO to Co will surely take place;
- the lowest equilibrium p_H2O_/p_H2_ ratio accompanying the reduction of Fe_2_O_3_ to Fe is 0.094, being 2.4 E5 times larger than the average experimental p_H2O_/p_H2_ ratio of 4 E-7 found above, i.e. the reduction of Fe_2_O_3_ to Fe will surely take place;
- the highest equilibrium p_H2O_/p_H2_ ratio accompanying the reduction of Al_2_O_3_ to Al is 2.31 E-14, being 1.7 E7 times smaller than the maximum experimental p_H2O_/p_H2_ ratio of 4 E-7 found above, i.e. the reduction of Al_2_O_3_ to Al will surely not take place.

**Table 1:** Equilibrium p_H2O_/p_H2_ values calculated for different reactions at different temperatures from standard thermochemical data^56^.

Reaction	800 K	900 K	1000 K
CoO + H_2_ = Co + H_2_O	50.6	40.5	33.5
3Fe_2_O_3_ + H_2_ = 2Fe_3_O_4_ + H_2_O	9.30 E4	9.67 E4	8.95 E4
Fe_3_O_4_ + H_2_ = 3FeO + H_2_O	4.36	8.48	13.4
FeO + H_2_ = Fe + H_2_O	0.0944	0.135	0.179
ZnO + H_2_ = Zn + H_2_O	4.83 E-5	3.00 E-4	1.26 E-3
Al_2_O_3_ + 3H_2_ = 2Al + 3H_2_O	1.97 E-18	3.46 E-16	2.31 E-14

The reduction of ZnO should be considered separately, as it is a macroscopic substrate and not a 10 nm thin coating. As follows from Table 1, the equilibrium p_H2O_/p_H2_ ratio accompanying the reduction of ZnO to Zn increases from 4.83 E-5 at T = 800 K to 1.26 E-3 at T = 1000 K. As explained above, during the reduction step 0.062 mol of hydrogen has passed the system over the AZO plate. Thus, to saturate it with water vapor according to the ZnO + H_2_ = Zn + H_2_O equilibrium, the amount of H_2_O in the gas should be 0.062 × 4.83 E-5 = 3.00 E-6 mol at T = 800 K and 0.062 × 1.26 E-3 = 7.81 E-5 mol at T = 1000 K. Thus, the same amount of ZnO was probably reduced on the surface of the AZO substrate. As the molar volume of ZnO is 14.5 cm^3^/mol, the thickness of reduced ZnO on the surface of 0.5 cm × 1 cm = 0.5 cm^2^ AZO is 0.87 microns at T = 800 K and 22.7 microns at T = 1,000 K. As the melting point of Zn is 419° C = 692 K, zinc as a reduction product will appear as a thin liquid layer on top of AZO. As its molar volume is 9.16 cm^3^/mol, it will form a liquid layer with thickness of 0.55 micron at T = 800 K and 14.3 micron at T = 1000 K. As metallic liquid Zn does not wet the remaining surface of AZO (especially that its surface is saturated by the inert Al_2_O_3_), liquid zinc will form separated liquid droplets on surface of AZO. As the thickness of liquid zinc is 55 times (T = 800 K) or even 1430 times (T = 1000 K) larger compared to the thickness of the reduced Fe-Co alloy, liquid Zn will certainly dissolve the Fe-Co nanoparticles^66^ and in this way their catalytic activity will disappear. This is the reason why it was impossible to grow CNTs on AZO without the protecting alumina layer and so without catalyst particles.

**Figure 9:**
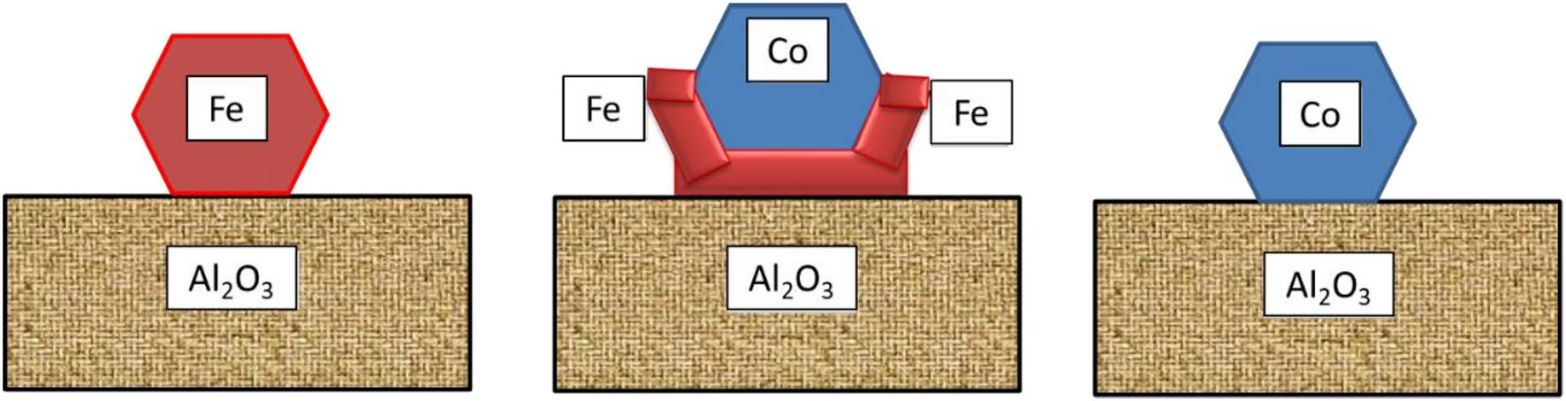
Predicted shape of Fe, Fe-Co alloy and Co nanoparticles on Al_2_O_3_ substrate.

However, the AZO substrate coated by the alumina layer will protect it from being reduced and so the Fe-Co nanoparticles will act as catalyst for the CNT growth. The equilibrium contact angle of molten Co and Fe droplets on Al_2_O_3_ substrate is around 120°^67–69^; although the Co-Fe grains are in solid state, this “contact angle concept” also applies to them in a way shown in Fig. 9.

### The structure and shape of Co-Fe nanoparticles on the Al_2_O_3_ substrate

According to the algorithm reported by Kaptay^70^, the surface energies of Co and Fe metals are nearly identical (the estimated difference is only 2.5%, with the surface energy of Co being slightly higher). This means that the bulk and surface compositions of Co-Fe nanoparticles in a reducing atmosphere are similar, and therefore no significant surface segregation is expected, at least not if there is only a single Fe-Co phase in the system.

**Figure 10:**
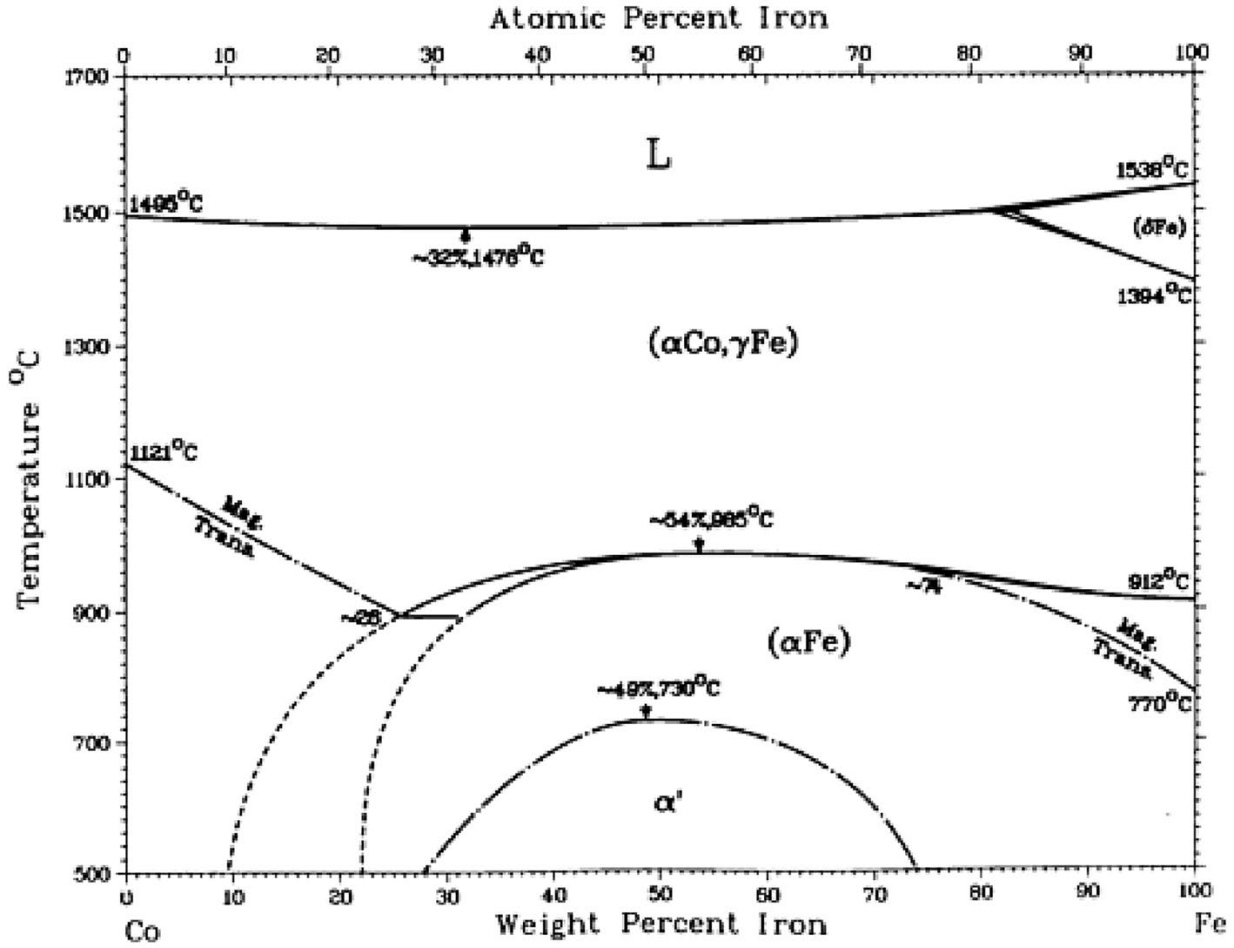
Equilibrium phase diagram of the Co-Fe alloy system^63^. Note: the equilibrium lines will shift down and to the middle by reducing the size, i.e. increasing the specific surface are of the system^71^.

Cobalt has a hcp lattice at room temperature and transforms to fcc lattice at 422 °C^66^. Iron has a bcc (ferromagnetic) lattice at room temperature, then transforms into a fcc (paramagnetic) lattice at 912 °C^66^. It follows from Fig. 10 that in the range of 600–700 °C the Fe-Co alloys with more than 23 at% Fe form bcc solid solutions^66^. The mole ratios used in the current work are: Fe:Co = 0:1, 1:3, 2:3, 1:1, 3:2, 3:1 and 1:0 meaning that apart from pure Co, all other samples are in the bcc solid solution state. However, Fig. 10 also shows that bcc solid solutions around equimolar composition undergo phase separation into Fe-rich and into Co-rich solid solutions with a critical (maximum) temperature around 730 °C^66^. Note that this critical temperature is probably somewhat reduced due to high specific surface area (not curvature) of the nanoparticles shown in Fig. 9 (see^71^).

In conclusion, except for pure Co and pure Fe particles, the Co–Fe alloys probably form two-phase structures: they contain a Co-rich bcc phase and an Fe-rich bcc phase, which presumably form a semi-coherent interface (their molar volume difference is about 7%—see^72^). For this reason, it is very likely that the Co–Fe grain will assume a core/shell structure. Since Fe has a lower surface energy, it is expected that the Fe-rich layer will be located on the outside, while the Co-rich layer is expected to be located on the inside.

Since the Co–Fe nanoparticle is situated on the surface of an Al_2_O_3_ substrate, their adhesion energy is also of interest. The component with stronger chemical affinity towards oxygen will have stronger adhesion energy with the oxide surface. According to Barin^56^, at 800–1000 K the heat of formation of CoO is around − 233 kJ/mol, while the same for FeO is around − 269 kJ/mol. Thus, not only because of its somewhat lower surface energy, but mostly because of its stronger adhesion energy to alumina, Fe becomes the interfacially active component at the Co–Fe/Al_2_O_3_ interface. Therefore, the Fe-rich bcc crystal will prefer the interfacial contact with Al_2_O_3_. Since the volumes of the Fe-rich and Co-rich phases are similar, it is expected that there will be a cup-shaped lower Fe-rich nanophase in contact with the oxide, embracing the Co-rich nanophase inside (see Fig. 9).

### The dissociation of ethylene and its reaction product

The standard molar Gibbs energy changes accompanying the dissociation of ethylene (C_2_H_4_) are given in Table 2 for two possible reactions at 3 temperatures of interest. One can see that the formation of 2C and 2H_2_ becomes more preferred at higher temperatures vs the formation of C + CH_4_. This is obviously due to entropy reasons: the first reaction produces more gaseous molecules than the second reaction from the same 1 mol of ethylene. Thus, above 818 K = 545 °C ethylene will preferably dissociate into graphite and hydrogen gas, the latter helps keeping the Fe–Co catalysts in the reduced state. Moreover, in this way twice more carbon atoms will be formed ready to build the CNTs further compared to the low-temperature case. However, this is an oversimplified analysis based solely on standard molar Gibbs energies. Now, let us improve it by considering the initial gas composition.

**Table 2:** Different molar Gibbs energy and enthalpy changes accompanying some chemical reactions (standard values are from^56^ for other values see text).

Reaction^a^	Quantity and its unit	800 K	900 K	1000 K
C_2_H_4_ (g) = 2C (gr) + 2H_2_ (g)	${\Delta }_{r}{G}_{H2}^{o}$, kJ/mol-C_2_H_4_	− 103	− 111	− 119
C_2_H_4_ (g) = C (gr) + CH_4_ (g)	${\Delta }_{r}{G}_{CH4}^{o}$, kJ/mol-C_2_H_4_	− 105	− 102	− 99
C_2_H_4_ (g) = 2C (gr) + 2H_2_ (g)	${\Delta }_{r}{G}_{H2}$, kJ/mol-C_2_H_4_	− 114	− 123	− 132
C_2_H_4_ (g) = C (gr) + CH_4_ (g)	${\Delta }_{r}{G}_{CH4}$, kJ/mol-C_2_H_4_	− 162	− 166	− 170
C(gr) = C(Fe)	solubility, at %	0.0025	0.013	0.090
C(gr) = C(Fe)	$\Delta {G}_{C(Fe)}^{E}$, kJ/mol-C	+ 70.5	+ 67.0	+ 58.3
C(gr) = C(Fe)	$\Delta {H}_{C(Fe)}$, kJ/mol-C	+ 96.1	+ 95.7	+ 87.5
C_2_H_4_ (g) = C (Fe) + CH_4_ (g)	${\Delta }_{r}{H}_{CH4}^{o}$, kJ/mol-C_2_H_4_	− 122.9	− 127.9	− 128.0
C_2_H_4_ (g) = C (Fe) + CH_4_ (g)	${\Delta }_{r}{H}_{CH4}$, kJ/mol-C_2_H_4_	− 26.8	− 32.2	− 40.5

^a^C(gr) means carbon in its standard state of pure graphite, C(Fe) means carbon dissolved in bcc-Fe.

As was shown in Chapter 2.3, during the CCVD process 50 Ncm^3^/min nitrogen + 50 Ncm^3^/min hydrogen + 70 Ncm^3^/min ethylene were added parallel into the reaction chamber for 5 min. This means that total 350 Ncm^3^ = 3.5 E-4 Nm^3^ ethylene passed the system, being equivalent to 0.014 mol of ethylene according to the ideal gas law. As follows from the experimental part, in the most successful experiment maximum 9 µm tall CNTs cover the 0.5 cm × 0.5 cm = 0.25 cm^2^ surface area of the substrate. Considering that only 5% of this volume is occupied by graphitic sheets^73^, the total volume of carbon about 1.15 E-5 cm^3^. Considering the 4.6 cm^3^/mol value of the molar volume of graphite, it means that about 2.5 E-6 mol of carbon was formed. It means that about 2.5 E-6 mol of ethylene dissociated, being less than 0.02% of the amount of ethylene that passed the system. Thus, the partial pressure of ethylene in the reaction chamber is about p_C2H4_ = 70/(70 + 50 + 50) = 0.41 bar, while the partial pressure of hydrogen is about p_H2_ = 50/(70 + 50 + 50) = 0.29 bar. If the dissociation of ethylene takes place with the formation of methane, then maximum 0.02% of the initial vapor pressure of ethylene is transformed into methane, so the maximum partial pressure of methane in the gas is p_CH4_ = 2 E-4 × 0.41 = 8.2 E-5 bar.

Now, let us calculate the molar (not standard) Gibbs energies of the dissociation reactions of ethylene using the above partial pressures values:

1a $${\Delta }_{r}{G}_{H2}={\Delta }_{r}{G}_{H2}^{o}+R\cdot T\cdot ln\left(\frac{{p}_{H2}^{2}}{{p}_{C2H4}\cdot {p}^{o}}\right) \quad \mathrm{for\,C_{2}}{\text{H}_4}({\text{g}}) = 2{\text{C}}({\text{gr}}) + 2{\text{H}_2}({\text{g}})$$

1b $${\Delta }_{r}{G}_{CH4}={\Delta }_{r}{G}_{CH4}^{o}+R\cdot T\cdot ln\left(\frac{{p}_{CH4}}{{p}_{C2H4}}\right) \quad \,\mathrm{for \, C_{2}} {\text{H}_{4}}({\text{g}}) =\mathrm{ C}({\text{gr}}) +\mathrm{ CH_{4}} ({\text{g}})$$

where R = 8.3145 J/molK is the universal gas constant, T (K) is absolute temperature, p^o^ = 1 bar, the standard pressure at which the standard molar Gibbs energy values used here are valid. Substituting the vapor pressures found above (p_C2H4_ = 0.41 bar, p_H2_ = 0.29 bar and p_CH4_ = 8.2 E-5 bar) into Eqs. (1a-b), the obtained values are given in rows 4–5 of Table 2. From their comparison one can see that ${\Delta }_{r}{G}_{CH4}\ll {\Delta }_{r}{G}_{H2}$, so we can conclude that the dissociation of ethylene takes place by the formation of methane. Note that this conclusion is opposite of what was claimed above based solely on standard molar Gibbs energy data. This is because during the process hydrogen is fed into the system, but methane is not. So, the driving force for this reaction is the lack of methane in the initial gas. Note that if amorphous carbon is formed, the dissociation of ethylene is a less favorable process, so this option is not considered here. Note also that the same result is obtained for the formation of pure graphite phase and for the carbon dissolved in a metallic phase saturated in carbon.

This latter comment is important, as the homogeneous nucleation of graphite in the gaseous phase is problematic. Therefore, dissociation of the ethylene gas has a higher probability on the surface of metals that can dissolve the carbon atoms and so nucleation of graphite does not hinder the dissociation of ethylene. Indeed, carbon has a measurable solubility in bcc iron as shown in the 6th row of Table 2. As was shown above, Co (in Fe-Co alloys) is also present in the form of bcc-Co. However, as bcc-Co does not exist as pure Co, solubility of carbon in bcc-Co is not known. On the other hand, based on Fig. 9, ethylene will much probably dissociate on the surface of Fe nanoparticles for all Fe-Co alloys studied here, as the Co nanoparticle is surrounded by the Fe nanoparticle and thus it shields the Co nanoparticle from the gas flow. Therefore, in this analysis our focus will be on the dissolution of carbon atoms on surface of Fe nanoparticles with their dissolution into Fe-bcc and their diffusion into the bulk of the Fe nanoparticles.

The limited solubility of graphite in bcc-Fe means that the partial excess molar Gibbs energy of carbon in bcc-Fe ($\Delta {G}_{C(Fe)}^{E}$, J/mol, with graphite as reference state) has a positive value estimated as:

2a $$\Delta {G}_{C(Fe)}^{E}\cong -R\cdot T\cdot {x}_{C(Fe)}^{max}$$

where ${x}_{C(Fe)}^{max}$ (dimensionless) is the maximum mole fraction of C in bcc-Fe that is found as 0.01 multiplied by solubility given in at% in Table 2. The values that follow from Eq. (2a) are given in the 7th row of Table 2. Based on the general rule developed for solid and liquid metallic alloys the partial heat of mixing of a component can be estimated from its partial excess Gibbs energy as^74^:

2b $$\Delta {H}_{C(Fe)}\cong \frac{\Delta {G}_{C(Fe)}^{E}}{1-\frac{T}{3000}}$$

The calculated values by Eq. (2b) are given in the 8th row of Table 2. As follows, the dissolution of graphite into bcc-Fe is a strongly endothermic process. This conclusion also supported by the Calphad assessment of the Fe–C system^75^. The non-standard molar enthalpy change accompanying the dissociation of ethylene into methane and carbon (${\Delta }_{r}{H}_{CH4}$, J/mol-C_2_H_4_) with carbon dissolving in Fe-bcc is calculated as:

2c $${\Delta }_{r}{H}_{CH4}={\Delta }_{r}{H}_{CH4}^{o}+\Delta {H}_{C(Fe)}$$

where ${\Delta }_{r}{H}_{CH4}^{o}$ (J/mol-C_2_H_4_) is the standard molar enthalpy change accompanying the dissociation of ethylene into methane and graphite. These enthalpy values are given in the last rows of Table 2. One can see that the dissociation of ethylene into methane and carbon dissolved in bcc-Fe is an exothermic process, heating the system. This is the reason why some authors presume that the metallic nanoparticles melt during this process^76^.

### Melting of the metallic nanoparticles

As was found in Table 2, the dissociation reaction of ethylene into methane and graphite is exothermic. Although the dissolution of carbon in Fe-bcc is endothermic, the resulting complex process is still somewhat exothermic (see last row of Table 2). Let us consider if the Fe nanoparticle can melt, considering only the heat obtained until the Fe nanoparticle is saturated in carbon. Let us perform the analysis at T = 1000 K to increase the probability of melting: in this way the starting temperature is closer to the melting point of iron and for this case the reaction is the most exothermic in Table 2.

Let us consider initially 1 mol of pure Fe at T = 1000 K. As follows from Table 2, maximum 0.090 at % of C can be dissolved in it at this temperature, meaning the dissolution of 9.0 E-4 mol of C. Then, from the last row of Table 2 the heat accompanying this process is − 40.5 E3 × 9.0 E-4 = − 36.5 J. On the other hand, the standard molar heat capacity of Fe-bcc at T = 1000 K and *p* = 1 bar is 54.4 J/molK^56^, so the exothermic heat found above is sufficient to heat the Fe nanoparticles only by 0.67 K in the stage of saturation of Fe by carbon. Thus, the Fe nanoparticles will not melt.

Let us note that after the CNTs nucleate and start growing, they will conduct heat away efficiently from the Fe nanoparticle. So, we can conclude that the Fe nanoparticle much probably will remain in its original bcc-state. This conclusion is not changed even if one takes into account the high specific surface area (not curvature) of the Fe nanoparticle, leading to some melting point depression of the Fe nanoparticle^72^. This conclusion is not changed either even if we take into account that during the growth of CNT through the top of the Fe nanoparticle the negative of the partial heat of mixing (− $\Delta {H}_{C(Fe)}$ = − 90 kJ/mol) is released. As the Fe nanoparticle has a low size and good heat conductivity, it will have an almost homogeneous temperature field inside, with a negligible temperature gradient. To our opinion, this negligible temperature gradient cannot be a reason for nucleation and growth of the CNTs, as claimed in some literature sources^77–80^.

### Nucleation of graphitic nanoparticles on top of the Fe nanoparticles

As follows from Fig. 9, the gas flow will come into contact with Fe nanoparticles from their side and top. Originally the gas is free of methane and the iron is also free of carbon, as it is coming not from steel industry, rather from thermal decomposition of chemically pure iron nitrate. Thus, the formation and dissolution of the first carbon atoms along the surface and inside of the Fe nanoparticles is driven by a much higher driving force than shown in Table 2. This will lead to the saturation of the Fe surface layer in carbon. Meanwhile, the carbon atoms will diffuse from the surface into the bulk of the Fe particle. However, solid state diffusion is a much slower process compared to gas flow or even gas diffusion, and so the Fe surface will become over-saturated in carbon. Let us also note that the transport of ethylene molecules to the top surface of the Fe nanoparticles will be faster compared to its sides (see Fig. 9), due to the roughness of the AZO/alumina plate, improved further by the presence of millions of Fe–Co nanoparticles. That is why the top surface of the Fe nanoparticles will be oversaturated in carbon atoms first and only later their side surface will be saturated. That is why nucleation of a graphitic phase will take place on the top side of the Fe nanoparticle shown in Fig. 9. First, a short cylindrical CNT will grow vertically up C-atom by C-atom thanks to C-atom release by the dissociating ethylene molecules. However, the opposing carbon atoms along the inner sides of the CNT cylinder are so close to each other (about 5 nm) that they will attract each other due to interfacial adhesion force^81^ and therefore a fullerene hemisphere is gradually formed closing the top of the CNT (this is confirmed experimentally in^82^). Once the CNT is closed by the fullerene hemisphere, it becomes inactive as a further site for C-atom attachment, i.e. the dissociation of ethylene molecules cannot take place here, so the CNTs cannot grow further along their top surface.

### Growth of carbon fibers and carbon nanotubes

The phase diagram of Co–C is not very informative for Co because bcc-Co does not exist, it is only stabilized in a solid solution by Fe. Our experimental results revealed that carbon fibers were grown on pure iron catalyst, but not on pure cobalt, or at least much less than on pure iron. Therefore, we can assume that the flux of C atoms into and out of the pure Fe crystal is significantly higher than the flux of C atoms into and out of the pure Co crystal at the same temperature. Therefore, ethylene gas will dissociate at a higher rate at the Fe/gas interface than at the Co/gas interface. Therefore, the growth of the carbon nanotube will also be at least 1 order of magnitude faster on the Fe/gas surface than on the Co/gas surface.

**Figure 11:**
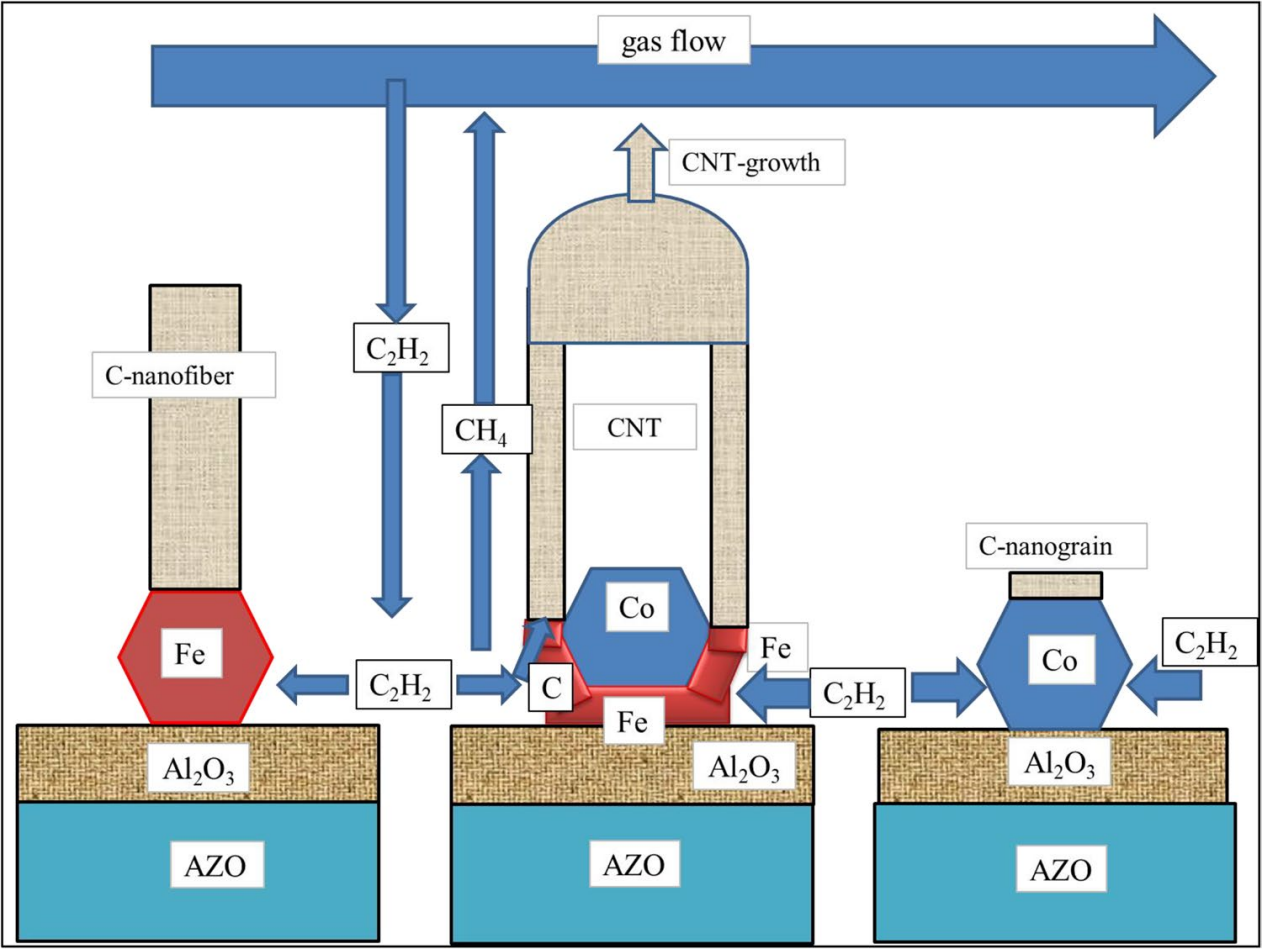
Carbon fiber and carbon nanotube growth on different metal nanoparticles in ethylene gas (schematic).

Considering the above results, we can predict the mechanism of carbon deposition using Fe, Fe–Co alloy and Co nanoparticles on Al_2_O_3_ substrate which were shown in Fig. 9. Now, it is quite clear why carbon nanofibers grow on pure Fe particles, why carbon nanobumps grow much more slowly on pure Co particles and why carbon nanotubes grow on Co-Fe two-phase nanoparticles (see Fig. 11).

Carbon is grown vertically from Fe nanoparticles because the dissociating ethylene molecules first oversaturate the top surface of Fe-nanoparticles and so nucleation and growth of the CNTs can start only on the top surface of iron particles, growing vertically up. When there is only one Fe phase, the growth of the carbon structure will not include an empty channel inside, so a carbon fiber is formed. However, when there are two phases as shown in Figs. 9 &11, then the carbon structure will grow much faster from the top Fe surface compared to the inside top Co surface, and therefore the carbon structure with a channel inside will be formed, called a carbon nanotube. Since the maximum temperature of Co–Fe bcc phase separation is 730 °C, it is expected that above 730 °C Co–Fe cannot catalyze carbon nanotube growth by this mechanism (see Fig. 10 and the comment in its caption). Short carbon fibers are grown slowly from pure Co, similarly as they do from pure Fe particles, although much faster in the latter case.

After the top of the short CNT is closed by the fullerene hemisphere, the CNT cannot grow by adding further C-atoms to its top. Instead, it can only grow by dissociating the ethylene molecules at the side surfaces of the Fe nanoparticles. As a result, carbon atoms will be dissolved along the sidewall of the Fe nanoparticle and they will diffuse towards the other end of the Fe nanoparticle, towards the roots of the CNT. In this way the CNT will grow further from its bottom, the new carbon atoms pushing it vertically upwards. The driving force of this process is about − 102 kJ/mol-C given in the 3rd row of Table 2 at T = 900 K, used as an example here. This is because the molar Gibbs energy change accompanying the dissolution of carbon along the sidewalls of the Fe nanoparticles will be compensated by the molar Gibbs energy change accompanying the precipitation of the same C atoms along the top surfaces of the Fe nanoparticles, at the roots of the CNTs. Moreover, to maintain the diffusion flux of ethylene molecules from the gas flow into the CNT forest and the diffusion flux of methane molecules from the CNT forest into the gas flow some driving force is needed taken equal in this first approximation the difference between ${\Delta }_{r}{G}_{CH4}$ and ${\Delta }_{r}{G}_{CH4}^{o}$ values shown in Table 2.

First, let us suppose that the sidewalls of the Fe nanoparticles will be saturated in the C atoms due to dissociation of the ethylene molecules at the Fe/gas interface and the rate-limiting step of CNT growth will be the diffusion of C atoms through the Fe nanoparticle. Thus, practically all the driving force of − 102 kJ/mol-C will be used to create and maintain a carbon concentration difference between the side and the top of the Fe nanoparticle. Then, the minimum molarity of C atoms in the Fe nanoparticle at its top (${C}_{C(Fe)}^{min}$) can be calculated as:

3a $${C}_{C(Fe)}^{min}\cong {C}_{C(Fe)}^{max}\cdot exp\left(\frac{{\Delta }_{r}{G}_{CH4}}{R\cdot T}\right)$$

where ${C}_{C(Fe)}^{max}$ (mol/m^3^) is the maximum molarity of C atoms along the side wall of the Fe nanoparticle. Substituting T = 900 K, ${\Delta }_{r}{G}_{CH4}$ = − 102 kJ/mol and ${C}_{C(Fe)}^{max}$ = 1.8 mol-C/m^3^ (= 1.3 E-4 mol-C/mol-Fe/7.1 E-6 m^3^/mol-Fe = the ratio of solubility of C in Fe-bcc from Table 2 divided by the molar volume of Fe-bcc) into Eq. (3a) we obtain: ${C}_{C(Fe)}^{min}$ = 2.2 E-6 mol-C/m^3^. If the distance between the side wall and the top side of the Fe nano-particle is d = 5 nm, then the concentration gradient of C-atoms within the Fe nano-particle is $\left({C}_{C(Fe)}^{min}-{C}_{C(Fe)}^{max}\right)/d$ = (2.2 E-6–1.8)/5 E-9 = -3 E8 mol-C/m^4^. Let us note that it is a huge concentration gradient of C atoms within the Fe nanoparticle. The diffusion flux of C atoms through the Fe nanoparticle (${F}_{C(Fe)}$, mol/m^2^s) is written after the first law of Fick as:

3b $${F}_{C(Fe)}\equiv \frac{{dn}_{C(Fe)}}{A\cdot dt}=-{D}_{C(Fe)}\cdot \frac{{C}_{C(Fe)}^{min}-{C}_{C(Fe)}^{max}}{d}$$

where ${D}_{C(Fe)}$ (m^2^/s) is the diffusion coefficient of C atoms in the Fe-bcc matrix at given temperature. Substituting the possible range of ${D}_{C(Fe)}$ = 1 E-12 … 1 E-11 m^2^/s^83^ and $\left({C}_{C(Fe)}^{min}-{C}_{C(Fe)}^{max}\right)/d$ = − 3 E8 mol-C/m^4^ into Eq. (3b): ${F}_{C(Fe)}$ = 3 E-4 … 3 E-3 mol/m^2^s is obtained. Multiplying this value by the molar volume of graphite (4.6 E-6 m^3^/mol), the linear growth rate of CNT is obtained as 1.4 E-9 … 1.4 E-8 m/s = 1.4 … 14 nm/s. Thus, during 30 min = 1800s of time about 2.5 … 25 microns long CNTs can grow, which is the same magnitude as found in our experiments, confirming the validity of this model.

Now, let us estimate the flux of C atoms in form of ethylene molecules through the gas phase (${F}_{C(gas)}$, mol/m^2^s):

3c $${F}_{C(gas)}\equiv \frac{{dn}_{C(gas)}}{A\cdot dt}=-{D}_{C(gas)}\cdot \frac{{C}_{C(gas)}^{min}-{C}_{C(gas)}^{max}}{L}$$

where ${D}_{C(gas)}$ (m^2^/s) is the diffusion coefficient of ethylene molecules in the gas phase at given temperature, L is the vertical length of the CNTs, equal the vertical diffusion length of the ethylene molecules from the bulk gas flow to the Fe nanoparticles. The value of ${C}_{C(gas)}^{max}$ follows from the partial pressure of ethylene calculated above (p_C2H4_ = 0.41 bar). Substituting this value and T = 900 K into the ideal gas law the molar volume of ethylene gas is 0.18 m^3^/mol and its inverse value is ${C}_{C(gas)}^{max}$ = 5.5 mol/m^3^. The maximum flux of carbon through the gas phase is obtained at ${C}_{C(gas)}^{min}\ll {C}_{C(gas)}^{max}$ maintained by the molar Gibbs energy difference discussed above. The diffusion coefficient of ethylene in the gas phase at T = 900 K is ${D}_{C(gas)}$ > 1 E-5 m^2^/s^84^. Substituting these values and ${F}_{C(gas)}$ = ${F}_{C(Fe)}$  = 3 E-4 … 3 E-3 mol/m^2^s into Eq. (3c), the minimum diffusion length of L > 0.02 m is estimated that can maintain the growth rate of CNTs as calculated above. As this value is higher by 3 orders of magnitude compared to the height of CNTs in our experiments we can conclude that the ethylene flux from the flowing bulk gas phase to the Fe nanoparticles is not a rate limiting step of CNT growth and it is sufficient to maintain the constant flux rate of C atoms for CNT growth of constant rate in our experiments as calculated above. The CNT growth rate can be decreased (or even stopped) only, if the surface of Fe nanoparticles is contaminated through the gas phase inhibiting the dissolution of C atoms into Fe-bcc and/or inhibiting the diffusion of C atoms through the Fe bcc nanoparticle (called “catalyst poisoning” in the literature^85–87^).

## Conclusion

Our experimental results presented in this communication have demonstrated that it is feasible to fabricate vertically aligned carbon nanotubes by CCVD onto AZO substrate. The experiments were carried out in the temperature range of 600–700 °C on 0.11 M iron-cobalt catalyst layers with different compositions (Fe:Co = 0:1, 1:3, 2:3, 1:1, 3:2, 3:1 and 1:0), formed by a simple dip coating method on the AZO surface with and without an alumina support layer. SEM images confirmed that carbon deposits were formed on the substrate surface without the alumina support layer at all three synthesis temperatures, but they were disorderly arranged. Our studies demonstrated that the Al_2_O_3_ support layer allowed for a more uniform catalyst particle distribution, whereby at 600 °C a significant amount of carbon nanotubes was observed in disordered form, while at 650 °C and 700 °C vertically aligned carbon nanotubes were successfully produced on AZO substrate. The experimental observations were supported step-by-step by theoretical considerations.

By the thermodynamic—transport analysis presented here the following is proven:

i. Co-nitrate, Fe-nitrate and Al-nitrate dissociates into Co-oxide, Fe-oxide and Al-oxide upon heat treatment below 400 °C.
ii. Co-oxide and Fe-oxide are reduced to Co and Fe in a hydrogen flow between 800 and 1000 K.
iii. Al-oxide remains intact, i.e. it is not reduced to Al in a hydrogen flow between 800 and 1000 K.
iv. The top layer of Zn-oxide is also reduced to Zn in a hydrogen flow between 800 and 1000 K; the resulting Zn melts and dissolves the Co and Fe nanoparticles, so their catalytic function is lost. This can be prevented if ZnO is protected by Al_2_O_3_.
v. The Co–Fe alloy forms a bcc solid solution at the temperature of CNT synthesis; this bcc solid solution undergoes phase separation below 730 °C to form an Fe-rich bcc solution and a Co-rich bcc solution. This two-phase Fe–Co alloy forms a core–shell structure on Al_2_O_3_ substrate with the Fe-rich shell phase in contact with Al_2_O_3_ substrate, and the Co-rich core phase surrounded along its lower part by the Fe-rich shell but being open upwards.
vi. Ethylene gas (fed together with some hydrogen gas) will dissociate into methane gas and graphite between 800 and 1000 K. However, nucleation of the graphite phase is possible only on the surface of a metal with good carbon solubility. Thus, ethylene will mostly dissociate on the surface of Fe nanoparticles and the resulting carbon atoms will be dissolved in the Fe nanoparticle. A similar process will take place but at a smaller rate on Co surface.
vii. Although dissociation of ethylene is an exothermic process, there will be a negligible temperature gradient within the Fe nanoparticle.
viii. The first graphite nanophase will nucleate on top surface of the Fe nanoparticle, as the transport of ethylene to this surface is faster compared to the side walls of Fe nanoparticles. This first graphite nucleus will be cylindrical (the hole in the cylinder is due to the Co-nanoparticle core in the shell of the Fe nanoparticle). This cylinder first will grow vertically up along its top surface C-atom by C-atom due to ethylene dissociation. As the distance between the opposing C-atoms in this graphitic cylinder is around 5 nm, the C-atoms will be attracted to each other by the interfacial adhesion force and therefore a fullerene hemi-sphere will cover the top of a short CNT. When this happens, the CNT will lose its nucleation activity, i.e. no more ethylene molecule can dissociate on its top surface.
ix. After the top of the CNT is closed by the fullerene hemisphere, the dissociation of ethylene will be possible only along the side walls of the Fe nanoparticles. The carbon atoms dissolved along the side walls of Fe nanoparticles will diffuse to the top of the Fe nanoparticle, i.e. to the roots of the CNTs and the CNTs will grow further up from its roots by adding further carbon atoms. There will be a huge concentration gradient of carbon atoms within the Fe nanoparticle with their maximum concentration along the side wall of the Fe nanoparticle and with their minimum concentration along the top surface of the Fe nanoparticle. i.e. at the root of the CNT. The linear vertical velocity of CNT growth is estimated from the carbon flux through the Fe nanoparticle as 1.4 … 14 nm/s, leading to total height of CNTs of about 2.5 … 25 µm during their production time of 30 min. This growth rate is confirmed by our experimental data.
x. It is shown that the diffusion flux of ethylene molecules from the bulk of the gas phase through the CNT forest to the Fe nanoparticle is much larger than the flux of C atoms through the Fe-bcc phase if the height of the CNT forest is below 0.02 m. As this is 3 orders of magnitude higher than our experimental results, it is concluded that the rate limiting step of CNT growth is the diffusion flux rate of carbon atoms through Fe nanoparticle and the diffusion of ethylene molecules through the gas phase has negligible influence on the latter.

## Data Availability

The datasets used and/or analyzed during the current study available from the corresponding author on reasonable request.
